# Supplementary material for: Complex‐centric proteome profiling by SEC‐SWATH‐MS
Source: Mol Syst Biol. 2019 Jan 14;15(1):e8438. doi: 10.15252/msb.20188438 (PMC6346213; doi:10.15252/msb.20188438)
Supplement: Supplementary file 6 — Dataset EV5 [file MSB-15-e8438-s006.zip › feature_plots_corum/2857.pdf]

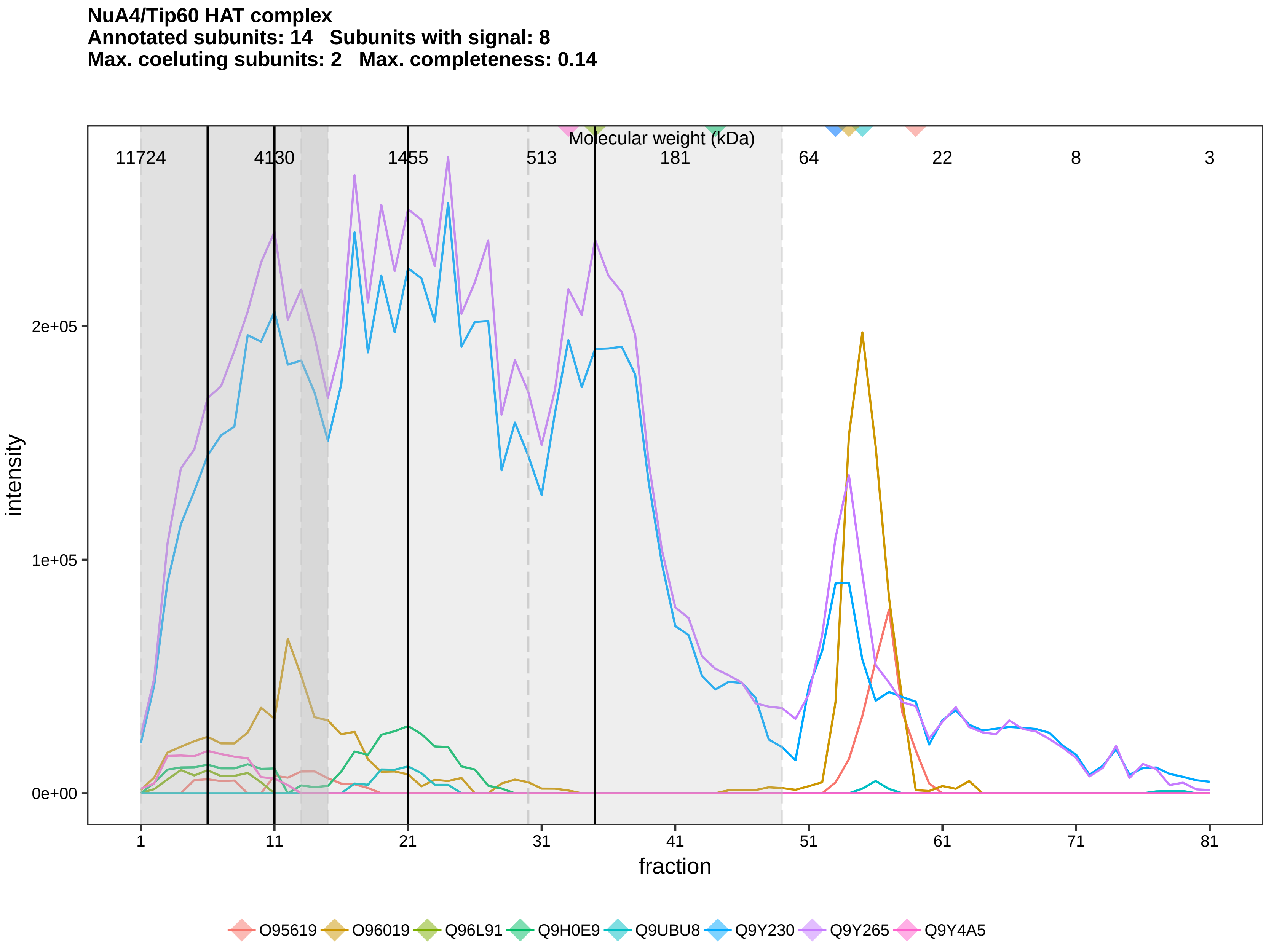
NuA4/Tip60 HAT complex
Annotated subunits: 14   Subunits with signal: 8
Max. coeluting subunits: 2   Max. completeness: 0.14
Molecular weight (kDa)
11724
4130
1455
513
181
64
22
8
3
intensity
2e+05
1e+05
0e+00
1
11
21
31
41
51
61
71
81
fraction
O95619
O96019
Q96L91
Q9H0E9
Q9UBU8
Q9Y230
Q9Y265
Q9Y4A5
